# Supplementary material for: Demographic causes and social consequences of adult sex ratio variation
Source: Nat Commun. 2026 Apr 6;17:4921. doi: 10.1038/s41467-026-71230-4 (PMC13233950; doi:10.1038/s41467-026-71230-4)
Supplement: Supplementary file 1 — Supplementary Information [file 41467_2026_71230_MOESM1_ESM.pdf]

Supplementary Material for

Demographic causes and social consequences of adult sex ratio variation

Authors: Zitan Song*, András Liker, Yang Liu, Robert P. Freckleton & Tamás Székely*

* Corresponding authors: songzitan@gmail.com; T.Szekely@bath.ac.uk

This PDF file includes:

Supplementary Figures 1 to 4
Supplementary Tables 1 to 12
Supplementary References

Supplementary Figures

Supplementary Figure 1.
Distribution of birth and adult sex ratios. Birth sex ratios (n = 103 species) and adult sex ratios (n = 261 species) are shown. Violin plots represent the distribution of sex ratios, with boxplots indicating the median and interquartile range. Horizontal dotted lines indicate sex ratios of 0.25, 0.5, and 0.75.

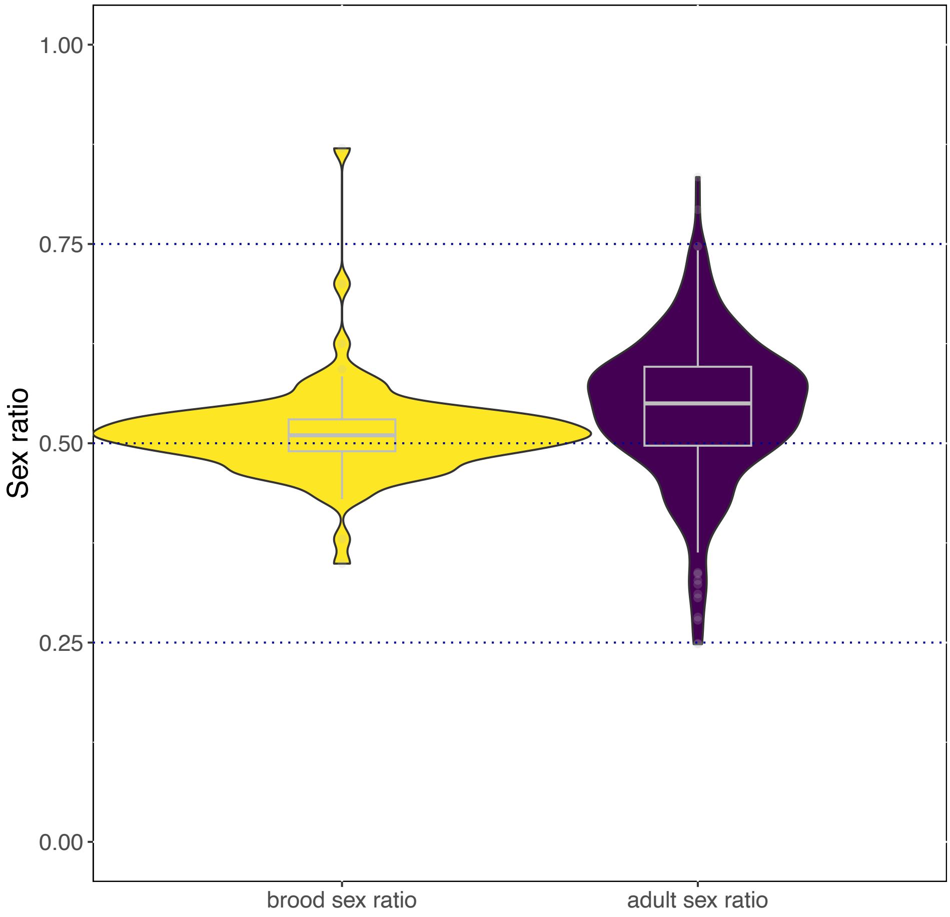

Supplementary Figure 2.
Path models tested in the phylogenetic path analyses with 65 species including both raw and imputed data. Models 1a-c and 2a-c represent relationships as predicted by the Figure 1, respectively.

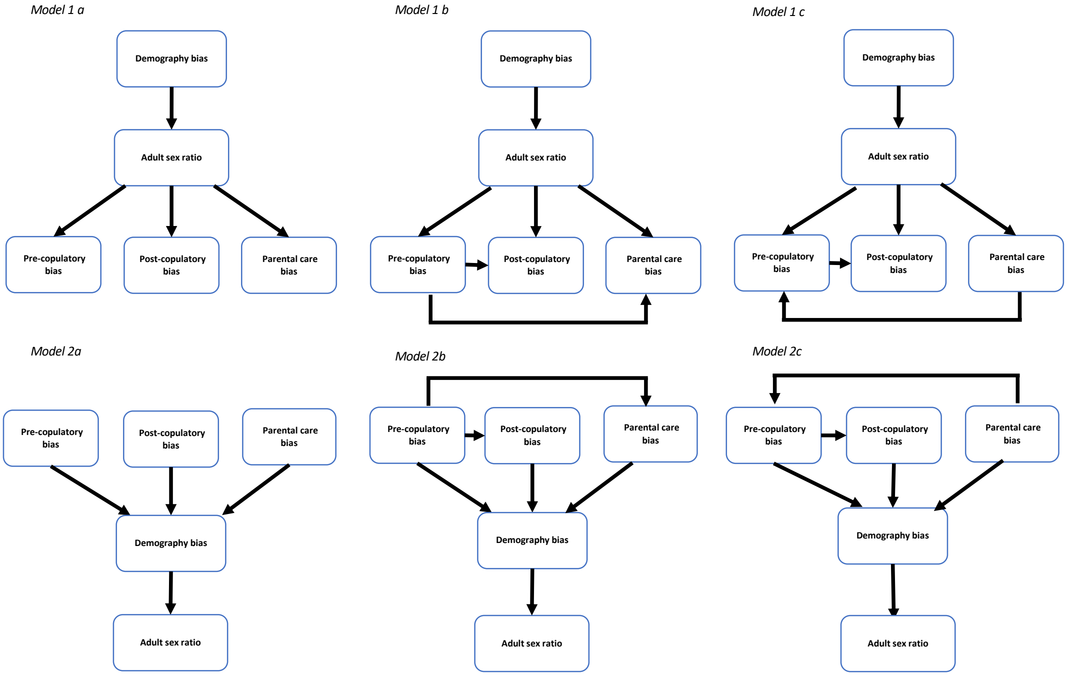

Supplementary Figure 3.
Path models tested in the phylogenetic path analyses with 67 species including only raw data. Models 1a-c and 2a-c represent relationships as predicted by the Figure 1, respectively.

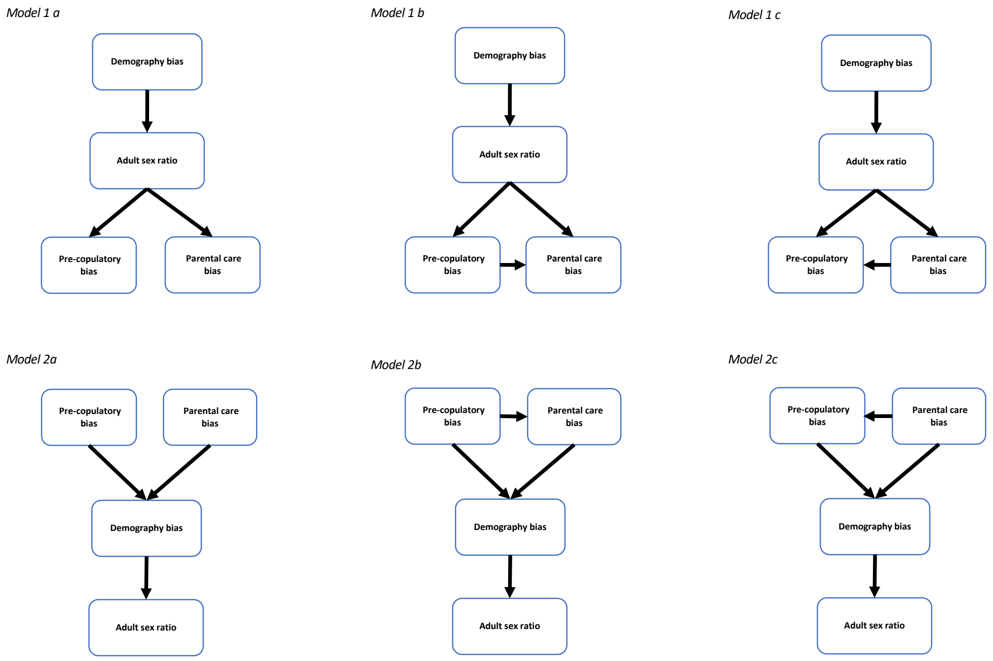

Supplementary Figure 4.
Best-supported phylogenetic path models for two model sets. (A) Best-supported model including post-copulatory bias (Supplementary Table 7, ΔAICc = 0.327). (B) Best-supported model excluding post-copulatory bias (Supplementary Table 8, ΔAICc = 0.093). Red lines indicate positive relationships and blue lines indicate negative relationships. Solid lines represent statistically significant paths, whereas dashed lines indicate non-significant relationships. Demographic bias is represented by the average difference between female and male juvenile mortality, adult mortality, and maturation delay. Pre-copulatory bias is represented by sexual size dimorphism, plumage dimorphism, and polygyny level. Post-copulatory bias is represented by relative testes mass and the level of extra-pair paternity. Parental care bias represents the difference between female and male parental investment.

A B

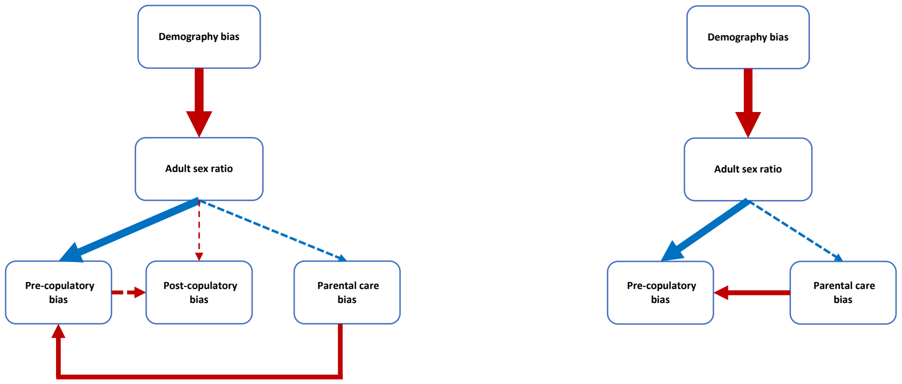

Supplementary Tables

Supplementary Table 1.
Principal Component Analyses (PCA) loadings and variance explained. Loadings of the main contributors to the different component are highlighted in bold.

a

Sex dimorphism in plumage	**PC1**
**Head**	**0.87**
**Back**	**0.90**
**Belly**	**0.84**
**Wings**	**0.81**
**Tail**	**0.78**
SS loading	3.54
Proportion Var	0.71

b

Female parental care	**PC1**
**Brooding (0-4)**	**0.87**
**Chick attend (0-4)**	**0.87**
SS loadings	1.52
Proportion Var	0.76

Supplementary Table 2.
Comparative analysis of adult sex ratios and four demographic variables in phylogenetically controlled MCMC models with **raw data** (n = 49 species). The analysis examines the relationship between ASR and brood sex ratio, maturation bias, and juvenile and adult mortality biases. Estimates are presented as posterior means with 95% posterior credibility intervals (CI); pMCMC denotes the MCMC-derived two-sided p value.

	Post. mean	lower – upper 95% CI	pMCMC
Intercept	0.524	0.494 – 0.559	0.0006
Birth sex ratio	0.015	-0.009 – 0.038	0.219
Juvenile mortality bias	0.045	0.018 – 0.068	0.001
Maturation bias	0.027	0.004 – 0.050	0.023
Adult mortality bias	0.026	0.002 – 0.053	0.050

Supplementary Table 3.
The ratio of the imputed data, the number of the species used for imputation, and the result of the reliability check. To increase the accuracy of the imputation co-variables were used, including: body mass (from[1]), development mode (precocial, semi- precocial, semi-altricial and altricial), development period (sum of incubation and fledging periods, main from[2], else from[1]), clutch size[1], male plumage colour [3], and local precipitation[3]. For the reliability check, each original (non-missing) data point was systematically removed once, re-imputed, and compared against it known value. The Pearson correlation coefficients are reported in the table.

Variable	The number of imputed data of dataset used	Variables used in the imputation	Correlation coefficients
Birth sex ratio (iBSR)	123/221	body mass, sexual size dimorphism, plumage dimorphism	0.058
Testes mass (iTestes)	80/235	body mass, mating system, clutch size, development mode; development period, local precipitation	0. 785
Extra-pair paternity (iEPP)	131/236	body mass, mating system, development mode, development period, male plumage color	0.567

Supplementary Table 4.

Comparative analysis of adult sex ratios and four demographic variables in phylogenetically controlled MCMC models with **imputed brood sex ratio data** (n = 67 species). The analysis examines the relationship between ASR and brood sex ratio, maturation bias, and juvenile and adult mortality biases. Estimates are presented as posterior means with 95% posterior credibility intervals (CI); pMCMC denotes the MCMC-derived two-sided p value.

	Post. mean	lower – upper 95% CI	pMCMC
Intercept	0.531	0.506 – 0.559	0.0006
Brood sex ratio (iBSR)	0.016	-0.003 – 0.032	0.082
Juvenile mortality bias	0.031	0.013 – 0.048	0.001
Maturation bias	0.035	0.017 – 0.052	0.002
Adult mortality bias	0.025	0.007 – 0.042	0.0006

Supplementary Table 5.
Influence of adult sex ratio on pre- and post-copulatory bias and parental care bias, and on associated traits including sexual size dimorphism, plumage dimorphism, mating system, relative testes mass, extra-pair paternity rate, and female-biased parental care in phylogenetically controlled MCMC models. Estimates are presented as posterior means with 95% posterior credibility intervals (CI); pMCMC denotes the MCMC-derived two-sided p value.

	Posterior mean	lower – upper 95% CI	pMCMC
**Pre-copulatory bias**			
**bivariate model[a]**			
Size dimorphism in mass (n = 254)			
Intercept	-0.035	-0.657 – 0.695	0.932
Adult sex ratio	-0.343	-0.424 – -0.250	0.0006
Plumage dimorphism[a] (PC1, n = 261)			
Intercept	-0.253	-0.997 – 0.477	0.502
Adult sex ratio	-0.095	-0.195 – 0.011	0.071
Mating system [a] (difference of polygamy between male and female, n = 248)			
Intercept	-0.231	-0.947 – 0.492	0.527
Adult sex ratio	-0.300	-0.407 – -0.200	0.0006
**Mean value from all three variables [a], n = 244**			
Intercept	-0.167	-0.723 – 0.447	0.551
Adult sex ratio	-0.277	-0.346 – -0.199	0.0006
**Post-copulatory bias**			
**bivariate model [a]**			
Relative testes mass (n = 164)			
Intercept	-0.223	-1.019 – 0.455	0.545
Adult sex ratio	-0.059	-0.198 – 0.085	0.440
Extra-pair brood (n = 109)			
Intercept	-0.323	-1.048 – 0.441	0.379
Adult sex ratio	0.029	-0.168 – 0.261	0.802
**bivariate model [b]**			
Relative testes mass (iTests, n = 226)			
Intercept	-0.271	-1.070 – 0.563	0.515
Adult sex ratio	-0.033	-0.145 – 0.077	0.563
Extra-pair brood (iEPP, n = 226)			
Intercept	-0.286	-1.091 – 0.424	0.456
Adult sex ratio	0.013	-0.079 – 0.110	0.814
**Mean value from all two variables [a] (n = 80)**			
Intercept	-0.097	-0.698 – 0.409	0.690
Adult sex ratio	-0.032	-0.232 – 0.174	0.750
**Mean value from all two variables [b] (iPCB, n = 226)**			
Intercept	-0.288	-0.88 – 0.314	0.346
Adult sex ratio	-0.017	-0.098 – 0.060	0.681
**Parental care bias**			
Sex-specific chick care PC1 (female participation in chick care (PC1) relative to male, n = 252) [a]			
Intercept	-0.188	-1.021 – 0.654	0.675
Adult sex ratio	-0.205	-0.288 – -0.120	0.0006

a: model built with raw data
b: model built with raw and imputation data

Supplementary Table 6.
Variance inflation factor (VIF) analyses (using the function *vif.phylolm*, see https://github.com/mrhelmus/phylogeny_manipulation) of all breeding system parameters against ASR.

**Parameter**	**VIF**
Pre-copulatory bias	1.674
Post-copulatory bias (iPCB)	1.072
Parental care bias	1.588

Supplementary Table 7.

Results of phylogenetic path analyses using imputed post-copulatory bias data (iPCB, n = 65 species). Model structures are shown in Supplementary Fig. 2. Fisher's C test statistic, degrees of freedom (df), exact two-sided P values, and model fit indices are reported as mean ± s.e. across phylogenetic trees. AICc, corrected Akaike information criterion; CFI, comparative fit index; TLI, Tucker–Lewis index; RMSEA, root mean square error of approximation; SRMR, standardized root mean square residual.

	Ficher C	df	P	AICc	cfi	tli	rmsea	srmr
Model 1a	25.128 ± 0.120	12	0.015 ± 0.001	55.246 ± 0.120	1.000 ± 0.000	1.097 ± 0.002	0.000 ± 0.000	0.022 ± 0.0004
**Model 1b**	**3.899 ± 0.080**	**8**	**0.859 ± 0.007**	**40.470 ± 0.080**	**1.000 ± 0.000**	**1.097 ± 0.002**	**0.000 ± 0.000**	**0.022 ± 0.0004**
**Model 1c**	**4.226 ± 0.087**	**8**	**0.829 ± 0.008**	**40.797 ± 0.087**	**1.000 ± 0.000**	**1.097 ± 0.002**	**0.000 ± 0.000**	**0.022 ± 0.0004**
Model 2a	22.281 ± 0.095	6	0.001 ± <0.0001	40.899 ± 0.095	0.683 ± 0.001	0.261 ± 0.003	0.303 ± 0.001	0.094 ± 0.0002
Model 2b	26.906 ± 0.121	8	0.001 ± <0.0001	63.477 ± 0.121	0.753 ± 0.001	0.383 ± 0.003	0.258 ± 0.001	0.096 ± 0.0002
Model 2c	25.937 ± 0.111	8	0.001 ± <0.0001	62.508 ± 0.111	0.753 ± 0.001	0.383 ± 0.003	0.258 ± 0.001	0.096 ± 0.0002

Supplementary Table 8.

Results of phylogenetic path analyses using raw data (n = 67 species). Model structures are shown in Supplementary Fig. 3. Fisher's C test statistic, degrees of freedom (df), exact two-sided P values, and model fit indices are reported as mean ± s.e. across phylogenetic trees. AICc, corrected Akaike information criterion; CFI, comparative fit index; TLI, Tucker–Lewis index; RMSEA, root mean square error of approximation; SRMR, standardized root mean square residual.

	Ficher C	df	P	AICc	cfi	tli	rmsea	srmr
Model 1a	18.239 ± 0.101	6	0.006 ± 0.0002	39.453 ± 0.101	1.000 ± 0.0001	1.018 ± 0.001	0.003 ± 0.001	0.027 ± 0.0003
**Model 1b**	**3.624 ± 0.047**	**4**	**0.463 ± 0.007**	**27.624 ± 0.047**	**1.000 ± 0.0001**	**1.018 ± 0.001**	**0.003 ± 0.001**	**0.027 ± 0.0003**
**Model 1c**	**3.717 ± 0.053**	**4**	**0.450 ± 0.007**	**27.717 ± 0.053**	**1.000 ± 0.0001**	**1.018 ± 0.001**	**0.003 ± 0.001**	**0.027 ± 0.0003**
Model 2a	24.535 ± 0.085	4	0.000 ± 0.000	40.466 ± 0.085	0.638 ± 0.001	0.094 ± 0.003	0.395 ± 0.001	0.124 ± 0.0003
Model 2b	26.512 ± 0.091	4	0.000 ± 0.000	50.512 ± 0.091	0.707 ± 0.001	0.121 ± 0.003	0.395 ± 0.001	0.124 ± 0.0003
Model 2c	25.566 ± 0.082	4	0.000 ± 0.000	49.566 ± 0.082	0.707 ± 0.001	0.120 ± 0.003	0.395 ± 0.001	0.124 ± 0.0003

Supplementary Table 9.
Results of phylogenetic path analyses conducted using the *phylopath* package. Each candidate model was evaluated using Fisher's C statistic and ranked by the C-statistic information criterion corrected for small sample size (CICc). Exact two-sided P values are reported. Model structures are shown in Supplementary Fig. 2 and Supplementary Fig. 3.

Models	k	q	Fisher's C	p	CICc	Delta CICc	weight
PPA with imputation post-copulatory data (iPCB, n=65)							
**Model 1c**	**4**	**11**	**3.212**	**0.920**	**30.193**	**0.000**	**0.586**
**Model 1b**	**4**	**11**	**3.911**	**0.865**	**30.892**	**0.699**	**0.413**
Model 2c	4	11	16.049	0.042	43.030	12.837	0.001
Model 2b	4	11	18.011	0.021	44.992	14.799	0.0004
Model 1a	6	9	24.516	0.017	45.789	15.596	0.0002
Model 2a	6	9	34.863	0.0005	56.136	25.943	<0.0001
PPA with raw data (n=67)							
**Model 1c**	**2**	**8**	**2.898**	**0.575**	**21.381**	**0.000**	**0.591**
**Model 1b**	**2**	**8**	**3.641**	**0.457**	**22.123**	**0.743**	**0.407**
Model 2c	2	8	15.767	0.003	34.249	12.868	0.001
Model 1a	3	7	18.628	0.005	34.526	13.145	0.001
Model 2b	2	8	17.624	0.001	36.107	14.726	0.0004
Model 2a	3	7	31.469	<0.0001	47.368	25.987	<0.0001

k= number of independence claims; q= number of parameters

Supplementary Table 10.
Effects of sex-biased adult mortality and sex-biased lifespan on the adult sex ratio in the 16-species overlap, estimated using phylogenetically controlled MCMC models. Estimates are presented as posterior means with 95% posterior credibility intervals (CI); pMCMC denotes the MCMC-derived two-sided P value. See the "Data and code availability" section for details on the data sources. Adult lifespan bias is defined as log(male post-maturation lifespan) – log(female post-maturation lifespan).

	Posterior Mean	Lower – upper 95% CI	pMCMC
Intercept	0.540	0.490 – 0.586	0.0006
Adult mortality bias	0.191	0.062 – 0.315	0.002
Adult lifespan bias	0.041	-0.041 – 0.113	0.260

Supplementary Table 11.
Influence of migration status on adult sex ratio and demographic bias in phylogenetically controlled MCMC models. Estimates are presented as posterior means with 95% posterior credibility intervals (CI); pMCMC denotes the MCMC-derived two-sided P value. Demographic bias is computed as the mean of juvenile and adult mortality biases and maturation bias.

	Posterior mean	lower – upper 95% CI	pMCMC
**Demography bias**			
**Resident vs partially migratory and fully migratory (n = 67)**			
Intercept	0.021	-0.144 – 0.177	0.758
Migration (*resident vs partially*)	-0.055	-0.184 – 0.084	0.412
Migration (*resident vs fully*)	-0.025	-0.149 – 0.091	0.710
**Partially migratory vs fully migratory (n = 41)**			
Intercept	-0.041	-0.245 – 0.140	0.640
Migration (*partially vs fully*)	0.038	-0.072 – 0.158	0.502
**ASR**			
**Resident vs partially migratory and fully migratory (n = 261)**			
Intercept	0.545	0.502 – 0.582	0.0006
Migration (*resident vs partially*)	-0.022	-0.050 – 0.010	0.176
Migration (*resident vs fully*)	-0.011	-0.041 – 0.016	0.466
**Partially migratory vs fully migratory (n = 158)**			
Intercept	0.528	0.479 – 0.574	0.006
Migration (*partially vs fully*)	0.011	-0.020 – 0.045	0.502

Migration status was coded from AVONET[4] as three categories: resident, partially migratory, and fully migratory.

Supplementary Table 12.
Influence of sex-biased dispersal on adult sex ratio and demographic bias in phylogenetically controlled MCMC models. Estimates are presented as posterior means with 95% posterior credibility intervals (CI); pMCMC denotes the MCMC-derived two-sided P value. Dispersal bias was calculated as log10(male dispersal distance) − log10(female dispersal distance); positive values indicate male-biased dispersal.

	Posterior mean	lower – upper 95% CI	pMCMC
	**Demography bias (n =29)**		
Intercept	-0.044	-0.220 – 0.116	0.620
Dispersal bias	-0.083	-0.192 – 0.030	0.152
	**ASR (n =63)**		
Intercept	0.521	0.481 – 0.558	0.001
Dispersal bias	0.000	-0.023 – 0.023	1.000

Sex-specific dispersal distances (male and female) were extracted from two independent sources[5,6]. Because sex-biased dispersal distance data availability was limited, we could not distinguish natal from breeding dispersal. When both were reported, we calculated the mean distance across natal and breeding dispersal for each sex and used this average to derive the sex-bias metric; when only one type was available, that value was used.

Supplementary References:

1. Székely, T. *et al.* Sex roles in birds: influence of climate, life histories and social environment. *Dryad Dataset* https://doi.org/https://doi.org/10.5061/ dryad.fbg79cnw7 (2022) doi:https://doi.org/10.5061/ dryad.fbg79cnw7.
2. Sayol, F., Downing, P. A., Iwaniuk, A. N., Maspons, J. & Sol, D. Predictable evolution towards larger brains in birds colonizing oceanic islands. *Nat Commun* **9**, 2820 (2018).
3. Delhey, K., Dale, J., Valcu, M. & Kempenaers, B. Migratory birds are lighter coloured. *Curr Biol* **31**, R1511–R1512 (2021).
4. Tobias, J. A. *et al.* AVONET: morphological, ecological and geographical data for all birds. *Ecol Lett* **25**, 581–597 (2022).
5. Fandos, G. *et al.* Standardised empirical dispersal kernels emphasise the pervasiveness of long-distance dispersal in European birds. *Journal of Animal Ecology* **92**, 158–170 (2023).
6. Végvári, Z. *et al.* Sex-biased breeding dispersal is predicted by social environment in birds. *Ecol Evol* **8**, 6483–6491 (2018).
