# Supplementary material for: Demographic causes and social consequences of adult sex ratio variation
Source: Nat Commun. 2026 Apr 6;17:4921. doi: 10.1038/s41467-026-71230-4 (PMC13233950; doi:10.1038/s41467-026-71230-4)
Supplement: Supplementary file 2 — Reporting Summary [file 41467_2026_71230_MOESM2_ESM.pdf]

nature portfolio

Corresponding author(s): Zitan Song

Last updated by author(s): Mar 4, 2026

Reporting Summary

Nature Portfolio wishes to improve the reproducibility of the work that we publish. This form provides structure for consistency and transparency in reporting. For further information on Nature Portfolio policies, see our Editorial Policies and the Editorial Policy Checklist.

Statistics

For all statistical analyses, confirm that the following items are present in the figure legend, table legend, main text, or Methods section.

n/a	Confirmed	
☐	☒	The exact sample size (*n*) for each experimental group/condition, given as a discrete number and unit of measurement
☐	☒	A statement on whether measurements were taken from distinct samples or whether the same sample was measured repeatedly
☐	☒	The statistical test(s) used AND whether they are one- or two-sided<br>*Only common tests should be described solely by name; describe more complex techniques in the Methods section.*
☐	☒	A description of all covariates tested
☐	☒	A description of any assumptions or corrections, such as tests of normality and adjustment for multiple comparisons
☐	☒	A full description of the statistical parameters including central tendency (e.g. means) or other basic estimates (e.g. regression coefficient) AND variation (e.g. standard deviation) or associated estimates of uncertainty (e.g. confidence intervals)
☐	☒	For null hypothesis testing, the test statistic (e.g. *F*, *t*, *r*) with confidence intervals, effect sizes, degrees of freedom and *P* value noted<br>*Give P values as exact values whenever suitable.*
☐	☒	For Bayesian analysis, information on the choice of priors and Markov chain Monte Carlo settings
☒	☐	For hierarchical and complex designs, identification of the appropriate level for tests and full reporting of outcomes
☐	☒	Estimates of effect sizes (e.g. Cohen's *d*, Pearson's *r*), indicating how they were calculated

Our web collection on statistics for biologists contains articles on many of the points above.

Software and code

Policy information about availability of computer code

Data collection	No software was used for data collection
Data analysis	All statistical analyses were carried out in R v4.1.1 using the following packages: MCMCglmm (v2.36), phylopath (v1.3.1), Rphylopars (v0.3.10), piecewiseSEM (v2.3.0), lavaan (v0.6.20), rptR (v0.9.23), psych (v2.5.6), and ape (v5.8.1).

For manuscripts utilizing custom algorithms or software that are central to the research but not yet described in published literature, software must be made available to editors and reviewers. We strongly encourage code deposition in a community repository (e.g. GitHub). See the Nature Portfolio guidelines for submitting code & software for further information.

Data

Policy information about availability of data

All manuscripts must include a data availability statement. This statement should provide the following information, where applicable:

- Accession codes, unique identifiers, or web links for publicly available datasets
- A description of any restrictions on data availability
- For clinical datasets or third party data, please ensure that the statement adheres to our policy

Data availability
The data used in this study are available in Figshare (https://doi.org/10.6084/m9.figshare.31490812)79. Figshare repository includes the dataset used in the analyses and the phylogenetic trees used for the phylogenetic mixed models and figure generation.

Code availability
The code used for data preparation, statistical analyses, and figure generation is available in Figshare (https://doi.org/10.6084/m9.figshare.31490812)79.

Research involving human participants, their data, or biological material

Policy information about studies with human participants or human data. See also policy information about sex, gender (identity/presentation), and sexual orientation and race, ethnicity and racism.

Reporting on sex and gender	N.A.
Reporting on race, ethnicity, or other socially relevant groupings	N.A.
Population characteristics	N.A.
Recruitment	N.A.
Ethics oversight	N.A.

Note that full information on the approval of the study protocol must also be provided in the manuscript.

Field-specific reporting

Please select the one below that is the best fit for your research. If you are not sure, read the appropriate sections before making your selection.

☐ Life sciences ☒ Behavioural & social sciences ☐ Ecological, evolutionary & environmental sciences

For a reference copy of the document with all sections, see nature.com/documents/nr-reporting-summary-flat.pdf

Behavioural & social sciences study design

All studies must disclose on these points even when the disclosure is negative.

Study description	This study is a quantitative comparative phylogenetic analysis based on published datasets. We analysed demographic and behavioural data from 261 bird species across 69 families to examine associations between adult sex ratio (ASR) and breeding systems using Bayesian mixed models and phylogenetic path analysis. The dataset was compiled from previously published studies and represents species for which reliable demographic and behavioural information is available.
Research sample	The sample consists of 261 bird species from 69 avian families.
Sampling strategy	This study is based on a systematic literature search and data compilation approach rather than a predefined sampling scheme. We compiled a species-level database of adult sex ratios (ASR) by searching Google Scholar and Web of Science for peer-reviewed studies reporting ASR estimates in birds. Demographic and reproductive trait data (e.g., birth sex ratio, sex-specific juvenile and adult mortality, maturation age, parental care, and sexual dimorphism) were then extracted for these species from published sources and comparative databases. No statistical sample size calculation was performed because the study relies on existing datasets. The final sample size varies among analyses depending on data availability for each variable, with up to 261 species included in the dataset.
Data collection	All data were collected using computer-based searches on Google Scholar and Web of Science, focusing on peer-reviewed publications containing species-level information on adult sex ratio (ASR), sex-biased demography, and breeding traits. The database was constructed collaboratively by multiple contributors, most of whom were not involved in the statistical analyses or hypothesis testing, and thus were effectively blind to the study hypotheses.<br>All extracted data were digitized from published sources; no original field or laboratory data were collected. No human or animal handling was involved.
Timing	Data compilation for this study was completed by November 1, 2025. The dataset was assembled through literature searches and extraction of information from previously published studies. The underlying primary data originate from studies conducted between the 1980s and 2025.
Data exclusions	No data was excluded from analyses.
Non-participation	Not applicable. The study did not involve human or live animal participants; it relied entirely on published datasets.
Randomization	Not applicable. No experimental groups or live individuals were involved.

Reporting for specific materials, systems and methods

We require information from authors about some types of materials, experimental systems and methods used in many studies. Here, indicate whether each material, system or method listed is relevant to your study. If you are not sure if a list item applies to your research, read the appropriate section before selecting a response.

Materials & experimental systems

n/a	Involved in the study
☒	☐ Antibodies
☒	☐ Eukaryotic cell lines
☒	☐ Palaeontology and archaeology
☐	☒ Animals and other organisms
☒	☐ Clinical data
☒	☐ Dual use research of concern
☒	☐ Plants

Methods

n/a	Involved in the study
☒	☐ ChIP-seq
☒	☐ Flow cytometry
☒	☐ MRI-based neuroimaging

Animals and other research organisms

Policy information about studies involving animals; ARRIVE guidelines recommended for reporting animal research, and Sex and Gender in Research

Laboratory animals	Not applicable. The study did not involve laboratory animals.
Wild animals	No new animals were captured, handled, or observed for this study.
Reporting on sex	The study focuses on sex differences in demography and reproductive traits across bird species. All sex-specific data were extracted from the literature. No individual-level data were collected.
Field-collected samples	Not applicable. No samples were collected from the field. All data were compiled from published studies.
Ethics oversight	Not applicable. The study involved only literature-based comparative analyses and did not require ethical approval.

Note that full information on the approval of the study protocol must also be provided in the manuscript.

Plants

Seed stocks	N.A.
Novel plant genotypes	N.A.
Authentication	N.A.
